# Traffic noise and cardiovascular disease mortality: A risk assessment

**DOI:** 10.17179/excli2021-3391

**Published:** 2021-01-29

**Authors:** Tomoyuki Kawada

**Affiliations:** 1 Department of Hygiene and Public Health, Nippon Medical School, 1-1-5 Sendagi, Bunkyo-Ku, Tokyo 113-8602, Japan

## ⁯⁯

***Dear Editor,***

There is an adverse effect of traffic nighttime noise on cardiovascular disease (CVD) outcomes (Münzel et al., 2020[4]). Cai et al. (2021[1]) conducted a meta-analysis on the association between long-term traffic noise and subsequent mortality with special reference to CVD, and that there was a trend of association between road traffic noise and ischemic heart disease mortality. In contrast, there was no significant association between aircraft noise and CVD mortality, partly because of the small number of related studies. They recommended of conducting high-quality longitudinal studies to evaluate the association, and I want to present recent reports.

First, Héritier et al. (2017[2]) reported the association between three major transportation noises and CVD mortality, and the adjusted hazard ratio (HR) (95 % confidence interval [CI]) of aircraft noise per 10 dB increase of exposure for myocardial infarction mortality was 1.026 (1.004-1.048). In addition, the adjusted HRs (95 % CIs) of the 3^rd ^and 4^th^ quintiles of intermittency ratio at night against continuous noise exposure for CVD mortality were 1.019 (1.002-1.037) and 1.021 (1.003-1.038), respectively. The risk of aircraft noise for CVD mortality changed by intermittency, and the characteristic of nighttime noise seems important for conducting a risk assessment.

Regarding aircraft noise, Saucy et al. (2020[6]) showed that nighttime aircraft noise significantly contributed to the increase of acute CVD mortality. The adjusted odds ratio (95 % CI) of inhabitants with exposure of LAeq > 50 dB against exposure < 20 dB for nighttime CVD mortality was 1.44 (1.03-2.04), which was more pronounced for females, people living in areas with low background noise, and people living in buildings constructed before 1970. In addition, there was a dose-response relationship. The effect of long-term noise exposure on CVD mortality should also be specified by considering the existence of habituation to noise (Kawada, 2011[3]).

Second, Osborne et al. (2020[5]) speculated the mechanism of the association between transportation noise and subsequent CVD events. The adjusted HR (95 % CI) of noise exposure per 5 dBA increase for major adverse cardiovascular disease events was 1.341 (1.147-1.567), and higher noise exposure was significantly associated with increased metabolic activity of amygdala and arterial inflammation. They speculated that noise exposure accelerated the stress-associated limbic activity and vascular inflammation. The neurobiological mechanism linking traffic noise to CVD events should be verified by considering the characteristics of noise.

## Conflict of interest

The author declares no conflict of interest.
